# The complete chloroplast genome of *Humulus lupulus* cv. ‘Fubei-1’ (Rosales: Cannabaceae)

**DOI:** 10.1080/23802359.2021.1926352

**Published:** 2021-07-20

**Authors:** Guoping Wang, Congzhao Fan, Yuanjin Qiu, Yaqin Zhao, Jizhao Zhang, Hailiang Xin, Xiaojin Li

**Affiliations:** a Xinjiang Institute of Chinese Materia Medica and Ethnical Materia Medica, Urumqi, China; b School of Pharmacy, Second Military Medical University, Shanghai, China

**Keywords:** *Humulus lupulus*, chloroplast genome, phylogenetic analysis, Bayesian inference

## Abstract

Hop (*Humulus lupulus*) is a perennial plant with commercial values. Here, we reported the complete chloroplast genome for a local hop cultivar (*Humulus lupulus* cv. ‘Fubei-1’) from Xinjiang, China. The chloroplast genome is 153,614 bp long with an A + T-biased base composition, and contains a total of 113 gene species, including 79 protein-coding, 30 tRNA, and four rRNA gene species. Nineteen gene species are duplicated, and 18 gene species harbor one or two introns. Phylogenetic analysis revealed close relatedness among the three hop cultivars (‘Saazer’, ‘Hallertauer’, and ‘Fubei-1’).

Hop (*Humulus lupulus*) is a perennial dioecious plant within the family Cannabaceae (order Rosales), and is widely distributed in Africa, North America, Europe, and Asia (including China’s Gansu, Sichuan, and Xinjiang Provinces) (Zhou and Bartholomew 2003). This plant has a long history of cultivation for beer brewing, and many cultivars have been developed across its geographic ranges (Hadonou et al. 2004; Almaguer et al. 2014; Vergara et al. 2016). Recently, chloroplast DNA has proven effective for cultivar identification in many plant species (Intrieri et al. 2007; Enan and Ahmed 2016; Jena et al. 2019). To date, chloroplast genomes have been sequenced for only a couple of hop cultivars, i.e. *Humulus lupulus* cv. ‘Saazer’ (NC_028032; Vergara et al. 2016) and *Humulus lupulus* cv. ‘Hallertauer’ (MG573060; Islam and Hamme, unpublished). In the present study, we assembled the complete chloroplast genome for a local hop cultivar (*Humulus lupulus* cv. ‘Fubei-1’) from Xinjiang Uygur Autonomous Region, China, and further investigated its relationship with other taxa within the family Cannabaceae.

Fresh leaves of a single individual were collected from Altay City, Xinjiang Uygur Autonomous Region, China (88.2207°E, 48.009°N). A voucher specimen (no. 654301170808030LY) was deposited at the Herbarium of the Xinjiang Institute of Chinese Materia Medica and Ethnical Materia Medica. The genomic DNA was extracted using the DNeasy Plant Mini Kit (QIAGEN, Valencia, CA) following the manufacturer’s instructions, and stored at −80 °C for further analysis. The quantity and quality of extracted DNA were determined using a NanoDrop ND-1000 Spectrophotometer (Thermo Fisher Scientific, Waltham, MA) and agarose gel electrophoresis, respectively.

A total amount of 0.5 μg DNA was used for the DNA sequencing library preparation with TruSeq Nano DNA HT Sample Prep Kit (Illumina, San Diego, CA) following the manufacturer’s protocol. Briefly, genomic DNA sample was fragmented by sonication to a size of 350 bp. Then, DNA fragments were endpolished, A-tailed, and ligated with the full-length adapter for Illumina sequencing, followed by further PCR amplification. After that, the library was analyzed for size distribution with Agilent 2100 Bioanalyzer (Agilent Technologies, Santa Clara, CA) and quantified by real-time PCR. The clustering of the index-coded samples was performed on a cBot Cluster Generation System using HiSeq X PE Cluster Kit V2.5 (Illumina, San Diego, CA) according to the manufacturer’s instructions. After cluster generation, amplicons were pooled in equal amounts, and paired-end 2–150 bp sequencing was performed using the Illumina HiSeq X Ten platform at Xuan Chen Biological Technology Co., Ltd. (Shaanxi, China).

The genome sequence data that support the findings of this study are openly available in GenBank of NCBI at https://www.ncbi.nlm.nih.gov/ under the accession no. PRJNA715949. The associated BioProject, SRA, and Bio-Sample numbers are PRJN 715949, SUB9201162, and SAMN18388804, respectively.

After quality-trimming with CLC Genomics Workbench v10 (CLC Bio, Aarhus, Denmark), the raw sequencing reads were used to assemble the chloroplast genome with MITObim v1.9 (Hahn et al. 2013). The chloroplast genome of *Humulus yunnanensis* (MK423880) (Ling and Zhang 2019) was selected as the initial reference. Genome annotation was conducted in GENEIOUS R11.0.2 (Biomatters Ltd., Auckland, New Zealand) by aligning those of phylogenetically related species.

The chloroplast genome of *H. lupulus* cv. ‘Fubei-1’ was determined to be 153,614 bp long with a typical A + T-biased base composition (31.1% A, 18.8% C, 18.1% G, and 32.0% T). A set of 113 gene species were annotated, including 79 protein-coding, 30 tRNA genes, and four rRNA gene species. Gene duplication occurs in 19 of these gene species (*ndh*B, *rpl*2, *rpl*23, *rps*7, *rps*12, *rps*19, *ycf*1, *ycf*2, *trn*A-UGC, *trn*I-CAU, *trn*I-GAU, *trn*L-CAA, *trn*N-GUU, *trn*R-ACG, *trn*V-GAC, *rrn*4.5, *rrn*5, *rrn*16, and *rrn*23). In addition, 16 gene species (*atp*F, *ndh*A, *ndh*B, *pet*B, *pet*D, *rpl*2, *rpl*16, *rpo*C1, *rps*12, *rps*16, *trn*A-UGC, *trn*G-UCC, *trn*I-GAU, *trn*K-UUU, *trn*L-UAA, and *trn*V-UAC) harbored a single intron and two gene species (*clp*P and *ycf*3) harbored two introns.

To ascertain its relationship to those confamilial taxa, phylogenetic analysis was conducted based on the Bayesian analysis of chloroplast protein-coding genes for a panel of 17 taxa within the family Cannabaceae (Figure 1). Three taxa from the family Moraceae (i.e. *Broussonetia papyrifera*, *Ficus altissima*, and *Morus cathayana*) were included as outgroup taxa. DNA alignment was conducted in Geneious R11 (Biomatters, Co. Ltd., Auckland, New Zealand), and was then imported into TOPALi v2.5 (Milne et al. 2009) for the phylogenetic analysis with the implemented MrBayes v3.1.1 program (Ronquist and Huelsenbeck 2003) under the best-fit nucleotide substitution model ‘GTR + G+I’. As expected, the phylogenetic analysis indicates that the three hop cultivars (‘Saazer’, ‘Hallertauer’, and ‘Fubei-1’) are most closely related to one another than to the other congeneric or confamilial taxa.

**Figure 1.**
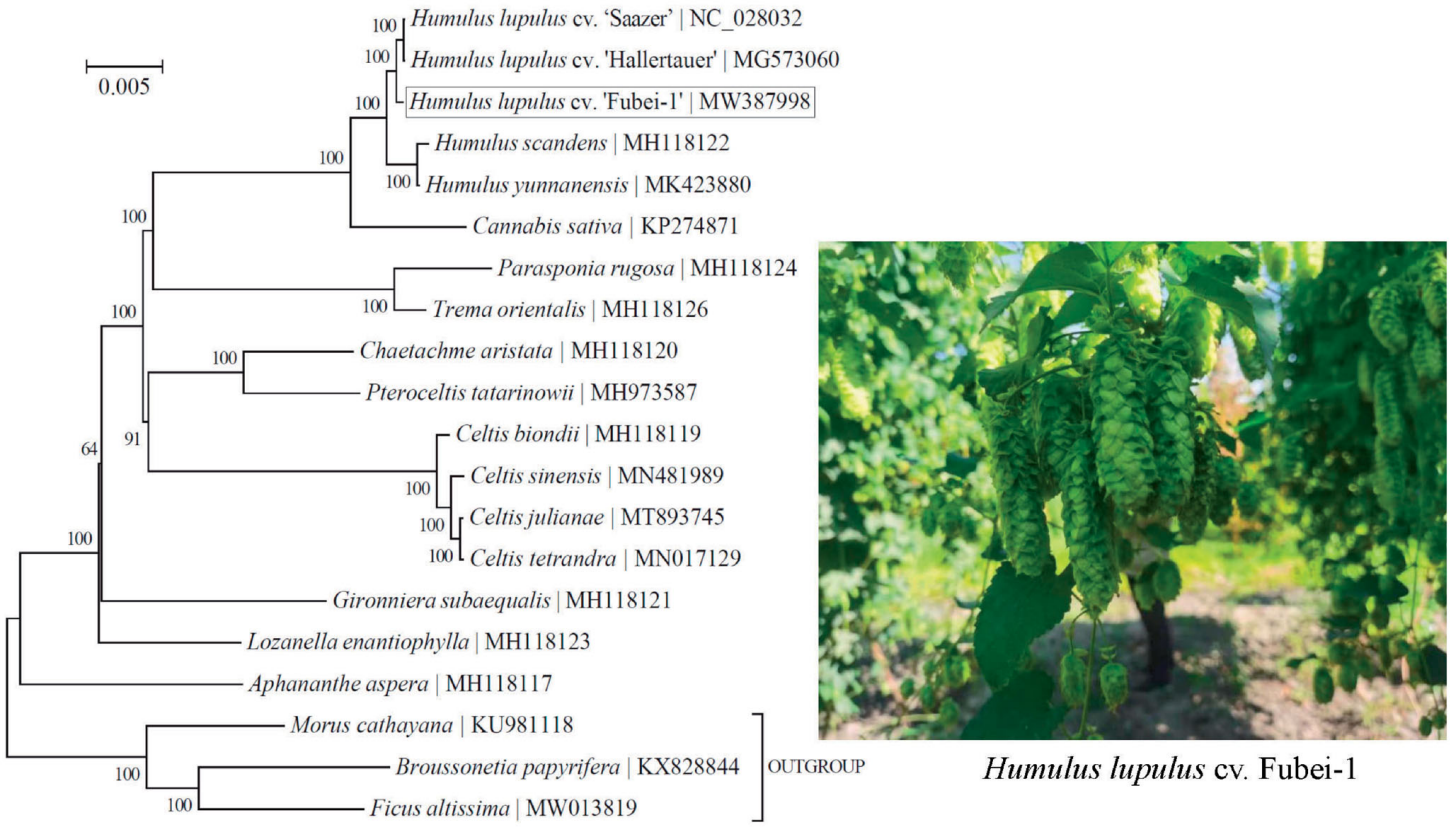
Phylogeny of 17 taxa within the family Cannabaceae based on the Bayesian analysis of chloroplast protein-coding genes. The best-fit nucleotide substitution model is ‘GTR + G+I’. The support values were placed next to the nodes. Three taxa within the family Moraceae (i.e. *Broussonetia papyrifera*, *Ficus altissima*, and *Morus cathayana*) were included as outgroup taxa.

## Data Availability

The data that support the findings of this study are openly available in GenBank from NCBI at https://www.ncbi.nlm.nih.gov under the accession number MW387998.
